# Factors associated with persistent positive in HBV DNA level in patients with chronic Hepatitis B receiving entecavir treatment

**DOI:** 10.3389/fcimb.2023.1151899

**Published:** 2023-06-16

**Authors:** Jun Li, Xiao-Qin Dong, Li-Hua Cao, Zhan-Qing Zhang, Wei-Feng Zhao, Qing-Hua Shang, Da-Zhi Zhang, An-Lin Ma, Qing Xie, Hong-Lian Gui, Guo Zhang, Ying-Xia Liu, Jia Shang, Shi-Bin Xie, Yi-Qi Liu, Chi Zhang, Gui-Qiang Wang, Hong Zhao, China HepB Related Fibrosis Assessment Research Group

**Affiliations:** ^1^ Department of Infectious Disease, Center for Liver Disease, Peking University First Hospital, Beijing, China; ^2^ Department and Institute of Infectious Diseases, Tongji Hospital, Tongji Medical College, Huazhong University of Science and Technology, Wuhan, Hubei, China; ^3^ Department of Hepatology, The Third Hospital of Qinhuangdao, Qinhuangdao, China; ^4^ Department of Infectious Disease, Shanghai Public Health Clinical Center, Fudan University, Shanghai, China; ^5^ Department of Infectious Disease, Xinxiang Medical University Affiliated Third Hospital, Xinxiang, China; ^6^ Department of Hepatology, No.88 Hospital of Chinese People’s Liberation Army (PLA), Jinan, China; ^7^ Department of Infectious Diseases, The Second Affiliated Hospital of Chongqing Medical University, Chongqing, China; ^8^ Department of Infectious Disease, China-Japan Friendship Hospital, Beijing, China; ^9^ Department of Infectious Diseases, Ruijin Hospital, School of Medicine, Shanghai Jiaotong University, Shanghai, China; ^10^ Department of Gastroenterology, The People’s Hospital of Guangxi Zhuang Autonomous Region, Nanning, China; ^11^ Department of Infectious Diseases, The Third People’s Hospital of Shenzhen, Shenzhen, China; ^12^ Department of Infectious Diseases, The People’s Hospital of Henan, Zhengzhou, China; ^13^ Department of Infectious Disease, The Third Affiliated Hospital of Sun-Yat Sen University, Guangzhou, China; ^14^ The Collaborative Innovation Center for Diagnosis and Treatment of Infectious Diseases, Zhejiang University, Hangzhou, Zhejiang, China; ^15^ Department of Hepatology, Peking University International Hospital, Beijing, China

**Keywords:** chronic hepatitis B, persistant viremia, HBV DNA, anti-hepatitis B virus core antibody, fibrosis, carcinoma

## Abstract

**Introduction:**

The clinical significance of persistent positive in Hepatitis B Virus (HBV) DNA level in patients receiving antiviral therapy is not well known. We investigated factors associated with persistent viremia (PV) in patients with chronic hepatitis B (CHB) given 78-week entecavir.

**Methods:**

A total of 394 treatment-naïve CHB patients who had undergone liver biopsy at baseline and week 78 of treatment were analyzed in this prospective multicentre study. We identified patients with PV (above the lower limit of quantification, 20 IU/ml) after 78 weeks of entecavir therapy. Stepwise, forward, multivariate regression analyses of specified baseline parameters were apllied to identify factors associated with PV. Futhermore, we assessed the incidence of hepatocellular carcinoma (HCC) in all patients using models of the risk of HCC development.

**Results:**

Of the 394 patients, 90 (22.8%) still with PV after 78-week antiviral treatment. Factors associated significantly with PV (vs complete virological response, CVR) were HBV DNA level ≥8 log10 IU/mL (OR, 3.727; 95% CI, 1.851-7.505; P < 0.001), Anti-HBc level < 3 log10 IU/mL (OR, 2.384; 95% CI, 1.223-4.645; P=0.011), and HBeAg seropositivity (OR, 2.871; 95% CI, 1.563-5.272; P < 0.001). Patients with PV were less likely to have fibrosis progression and HCC development than those with the CVR. Of the 11 HBeAg-positive patients with HBV DNA level ≥8 log10 IU/mL and Anti-HBc level < 3 log10 IU/mL at baseline, 9 (81.8%) had persistent positivity in HBV DNA level and 0 had fibrosis progression at week 78 of treatment.

**Discussion:**

In conclusion, HBV DNA level ≥8 log10 IU/mL, Anti-HBc level < 3 log10 IU/mL and HBeAg seropositivity at baseline contribute to PV in patients with CHB receiving 78-week antiviral treatment. In addition, the rate of fibrosis progression and the risk of HCC development in patients with PV were kept low. The complete protocol for the clinical trial has been registered at clinicaltrials.gov (NCT01962155 and NCT03568578).

## Introduction

Chronic hepatitis B (CHB) affects more than 250 million people worldwide and causes annual mortality of nearly 1 million from cirrhosis, hepatocellular carcinoma and other diseases associated with hepatitis B virus (HBV) infection (World Health Organization, 2016; Polaris Observatory Collaborators, 2018). Nucleos(t)ide analogues (NAs) approved for CHB treatment have been demonstrated to reduce HBV disease progression, reverse liver fibrosis and decrease the risk of hepatocellular carcinoma (HCC) development. Entecavir (ETV) and tenofovir disoproxil fumarate (TDF) are clinically used as first-line nucleos(t)ide analogue (NA) antivirals for the treatment of patients with CHB, and both have reasonably improved the rates of virological suppression, biochemical and serological response. Both drugs display high genetic barriers with very minimal resistance and high rates of viral suppression (Tenney et al., 2009; Chang et al., 2010; Yang et al., 2013).

In controlled clinical trials, most but not all patients with CHB experience undetectable serum HBV DNA levels on oral antiviral therapy. Reported rates of undetectable serum HBV DNA (<300 copies/mL) range from 67% to 97% among hepatitis B e antigen–positive (HBeAg+) patients (Chang et al., 2006; Gish et al., 2007; Pan et al., 2012; Marcellin et al., 2013) and 90% to 97% among HBeAg-negative (HBeAg-) patients (Lai et al., 2006; Marcellin et al., 2013). Precisely why a proportion of patients do not achieve an undetectable serum HBV DNA level despite apparently effective antiviral treatment has not been fully explored. Both the stability of the HBV covalently closed circular DNA (cccDNA) in the hepatocyte nucleus (Allweiss and Dandri, 2017), and the resistance of HBV to NAs (Sinn et al., 2011) are believed to be some mechanisms.

Some reports suggested that persistent positive in HBV DNA level after NA therapy was associated with a higher risk of hepatocellular carcinoma (HCC) occurrence and fibrosis progression (Kim et al., 2017; Sun et al., 2020). However, it had also been reported that low-level viremia (LLV, <2,000 IU/mL) during treatment was not a predictive factor for HCC and cirrhotic complications in patients with treatment-naïve CHB and good adherence to ETV treatment (Lee et al., 2020). And Lee et al. suggested that episodic LLV among untreated patients with compensated cirrhosis did not increase the risk of disease progression compared with maintained virological response status (Lee et al., 2022). As a result, it remains unclear whether it is more beneficial to continue original NAs or to switch/add another NA in order to prevent liver-related events including fibrosis progression and HCC development in persistent viremia (PV, above the lower limit of quantification, 20 IU/ml) patients. The AASLD 2018 hepatitis B guidance suggested that persons with persistent LLV on ETV or TDF monotherapy should continue monotherapy, but the quality and certainty of this evidence was very low (Terrault et al., 2018).

Therefore, the objectives of this study were to identify factors associated with PV and investigate whether patients with PV were associated with HCC development and fibrosis progression, so as to further explore the next treatment options for patients with PV, by analyzing data collected from a well-characterized cohort of CHB patients that have been treated with ETV for 78 weeks.

## Materials and methods

### Patients

This multi-center, prospective, longitudinal study included 780 Chinese treatment-naïve patients with CHB who were consecutively admitted from 24 teaching hospitals located in Mainland China between October 2013 and May 2021. Patients recruited in the cohort study were those with hepatitis B surface antigen (HBsAg) positive for at least 6 months and negative of other forms of chronic liver diseases (CLD), decompensate liver cirrhosis or HCC. Patients who had received treatments with either bicyclol or antiviral drugs within 26 weeks before the recruitment were excluded. The specific inclusion and exclusion criteria had been described previously (Deng et al., 2015). Demographic data were collected at baseline. Clinical data, including blood test results and liver stiffness measurement (LSM) were recorded at the time of liver biopsy at baseline and every 26 weeks of follow-up in local hospitals. Paired liver biopsies were performed at baseline and week 78. The study was approved by the Ethics Committee of Peking University First Hospital and the other 23 teaching hospitals. All patients gave informed consent for research use of their clinical data and liver biopsy specimens. All authors had access to the study data and reviewed and approved the final manuscript.

### Laboratory assessments

Blood specimens were routinely obtained on the same day of liver biopsy in local hospitals. Serum HBV DNA quantitation was detected at central laboratory using Roche COBAS TaqMan platform (lower limit of detection 20 IU/mL) according to manufacturer’s instructions. Levels of HBV serological markers (HBsAg/anti-HBs, HBeAg/anti-HBe) were measured by relevant Roche Elecsys^®^ assays (Roche Diagnostics, Penzberg, Germany). Quantitative detection of anti-hepatitis B virus core antibody (Anti-HBc) was performed using Sandwich enzyme-linked immunosorbent assays (Jia et al., 2014). The level of HBV DNA, HBsAg, and Anti-HBc were expressed as log_10_ IU/mL.

### Liver stiffness measurement

LSM was performed on fasting patients at baseline and week 78 using 1-dimensional ultrasound TE (FibroScan^®^, Echosens, Paris, France) in local hospitals. All operators who had no knowledge of the patients’ clinical data were trained according to the manufacturer’s recommendations. LSM values are expressed in units of kilopascals (kPa). Only a procedure with at least ten valid measurements, an interquartile range (IQR)/median value (IQR/M) <30% and a success rate >60% was considered reliable (Sandrin et al., 2003).

### Fibrosis-4 and AST to platelet ratio index scores

The two noninvasive indexes for fibrosis were calculated at baseline and week 78 based on the following formulas:


$$\text{FIB}-4=(\text{age}\times \text{AST})/(\text{platelet count})/[\times 10^{9}/\text{L}]\times \text{AL}{\text{T}}^{1/2})$$

(Sterling et al., 2006);


$$\text{APRI}=([\text{AST}/\text{ULN}]/\text{platelet count}[\times 10^{9}/\text{L}])\times 100$$

(Wai et al., 2003).

### Liver histological assessment

Ultrasonographic-guided liver biopsies were performed at baseline (on day before starting antiviral therapy) and week 78 in each institute. A minimum of 20 mm of liver biopsy with at least 11 portal tracts was considered adequate for diagnosis. All liver biopsies were blindly and independently reviewed by 2 hepatopathologists from Beijing You An Hospital affiliated to Capital Medical University. When discrepancies occurred, final decision was made by the third experienced hepatopathologist who was also responsible for reassessment of 10% samples by random drawing. Necro-inflammation and fibrosis were assessed with the Ishak scoring system (Ishak et al., 1995). Fibrosis was scored as follows: F0-1, no/mild fibrosis; F≥2, moderate fibrosis; F≥3, significant fibrosis; F≥4, advanced fibrosis; and F≥5, cirrhosis. Histological inflammation grading was performed using the modified histology activity index (HAI), and the scored as follows: HAI 0-4, zero to mild inflammation; HAI 5-18, moderate to severe inflammation. Histological improvement was defined as ≥2-point decrease in the HAI score and without concurrent worsening of the fibrosis score 78 weeks after the therapy initiation. Fibrosis improvement was defined as ≥1-point decrease in the Ishak fibrosis score, whereas ≥1-point increase was considered as fibrosis progression. Inflammation improvement was defined as ≥2-point decrease in the HAI score.

### Statistical analyses

Statistical analysis was performed using SPSS 20.0 (SPSS Inc., Chicago, IL, USA). Patients’ characteristics were summarized as median (range), or numbers of cases and percentages, as appropriate. Continuous variables were compared using Student t test or Mann-Whitney test, categorical variables were compared using Chi-squared test or Fisher’s exact test. Univariate and multivariate logistic regression analysis were used to identify independent predictors associated with persistent positivity in HBV DNA level after 78-week antiviral therapy. Factors significant in univariate analyses were included in the multivariate model using the forward selection procedure; predictors were retained in the model if the *P* value was less than 0.10. All statistical tests were two-sided, and *P*<0.05 was considered statistical significance.The original data was shown in https://github.com/Xiaoqind/Factors-associated-with-PV.

## Results

### Study population

Of 780 Chinese treatment-naïve CHB patients who met the eligible criteria and with qualified liver biopsy at baseline, 504 (64.6%) patients received entecavir-based therapy and were prospectively followed to 78 weeks for second liver biopsy. Ishak fibrosis scores was available at all two time-points (baseline and week 78) for 394 patients (**Supplementary Figure 1**). In total, 90 patients (22.8%) had PV at week 78.

### Predictors of persistent positive in HBV DNA level after 78-week antiviral therapy

Baseline demographic and disease characteristics of patients with PV and patients with complete virological responses (CVR, below the lower limit of quantification, 20 IU/ml) at week 78 are summarized in **Table 1**. Compared with the group that achieved CVR, patients with PV were younger. A higher proportion of patients with PV were HBeAg positive and this group had higher baseline HBV DNA and HBsAg levels, and lower Anti-HBc level compared with the CVR group. Baseline liver biopsy analysis showed that patients with PV had less fibrosis than patients with CVR (Ishak fibrosis score>3: 43.3% vs 56.6%; *P*=0.027), but the prevalence of necroinflammation was similar (HAI ≥5: 77.8% vs 70.7%; *P*=0.189). There was no difference between the two groups in the terms of sex, BMI, PLT, ALT, ALB, TBIL, AFP, CR, FPG, FIB-4, APRI, LSM, patients with family history of HCC or hepatitis B.

**Table 1:** Baseline demographics and disease characteristics of 394 patients with and without complete virological response at week 78 (univariate analysis).

Variables	Total n=394	Persistent viremia n=90	Complete virological response n=304	*P*-value*
Age (years) ≤30y, n (%)	77 (19.5)	27 (30.0)	50 (16.4)	**0.004**
Male gender, n (%)	294 (74.6)	69 (76.7)	225 (74.0)	0.611
BMI ≥25 kg/m^2^, n (%)	134 (34.0)	32 (35.6)	102 (33.6)	0.725
PLT (×10^9^/L)	155.0 (121.0-194.0)	155.0(127.3-199.0)	155.0 (118.3-193.8)	0.609
ALT ≤ 40 IU/ml, n (%)	123 (31.2)	24 (26.7)	99 (32.6)	0.289
ALT ≤ 30 IU/ml for male and ≤ 19 IU/ml for female, n (%)	47 (11.9)	8 (8.9)	39 (12.8)	0.311
Albumin ≥35 g/L, n (%)	373 (94.7)	88 (97.8)	285 (93.8)	0.135
TBIL ≤ 17.1 μmol/L, n (%)	245 (62.2)	53 (58.9)	192 (63.2)	0.463
TC ≤ 5.18 mmol/L, n (%)	332 (84.3)	71 (78.9)	261 (85.9)	0.111
TG ≤ 1.69 mmol/L, n (%)	351 (89.1)	81 (90.0)	270 (88.8)	0.752
Cr ≤ 106 IU/ml for male and ≤ 97 IU/ml for female, n (%)	387 (98.2)	88 (97.8)	299 (98.4)	0.661
FPG ≥7.0 mmol/L, n(%)	11 (2.8)	1 (1.1)	10 (3.3)	0.461
HBV DNA ≥8 log^10^IU/mL, n(%)	43 (10.9)	25 (27.8)	18 (5.9)	**<0.0001**
HBsAg ≥4 log10 IU/mL, n(%)	53 (13.5%)	27 (30.0)	26 (8.6)	**<0.0001**
HBeAg positive, n (%)	226 (57.4%)	73 (81.1)	153(50.3)	**<0.0001**
qAnti-HBc<3log^10^IU/mL), n(%)	49 (12.4%)	22 (24.4)	27 (8.9)	**<0.0001**
FIB-4	1.5 (1.0-2.3)	1.4 (0.9-2.3)	1.6 (1.0-2.4)	0.329
<1.45, n(%)	166 (42.1)	44 (48.9)	122 (40.1)	
1.45-3.25, n(%)	191 (48.5)	39 (43.3)	152 (50.0)	
>3.25, n(%)	37 (9.4)	7 (7.8)	30 (9.9)	
APRI	0.7 (0.4-1.3)	0.9 (0.4-1.4)	0.7 (0.4-1.3)	0.636
≤0.5, n(%)	105 (26.6)	23 (25.6)	82 (27.0)	
0.5-1.5, n(%)	186 (47.2)	40 (44.4)	146 (48.0)	
>1.5, n(%)	103 (26.1)	27 (30.0)	76 (25.0)	
LSM (kPa)	11.7 (8.1-17.6)	12.2 (8.6-19.0)	11.5 (7.8-17.2)	0.774
<7.4, n(%)	76 (19.3)	15 (17.4)	61 (20.1)	
7.4-9, n(%)	57 (14.5)	12 (13.0)	45 (14.8)	
9-12, n(%)	78 (19.8)	17 (17.8)	61 (20.1)	
≥12, n(%)	183 (46.4)	46 (41.8)	137 (45.1)	
HAI ≥5, n(%)	285 (72.3)	70 (77.8)	215 (70.7)	0.189
Fibrosis stages >3, n(%)	211 (53.6)	39 (43.3)	172 (56.6)	**0.027**
HAI ≥5 or F≥3, n(%)	365 (92.6)	82 (91.1)	283 (93.1)	0.527
Patients with family history of HCC, n(%)	48 (12.2)	14 (15.6)	34 (11.2)	0.270
Patients with family history of hepatitis B, n(%)	188 (47.7)	46 (51.1)	142 (47.0)	0.495

*P* value: comparision between patients with with and without complete virological response at week 78. %, percentage, range from 0-100%。

BMI, body mass index; ALT, Alanine transaminase; TBil, total bilirubin; CR, creatinine; FPG, fasting plasma glucose; PLT, platelet counts; TC total cholesterol; TG triglyceride; AFP, alpha fetoprotein; LSM, liver stiffness measurement; HBsAg, hepatitis B surface antigen; HBeAg, hepatitis B e antigen; HBV, hepatitis B virus; Anti-HBc, hepatitis B core antibody; HAI, histology activity index; ULN upper limit of normal; FIB-4, fibrosis-4; APRI, AST to platelet ratio index. The meaning of the bold values is to highlight statistically significant indicators.

**Figure 1:**
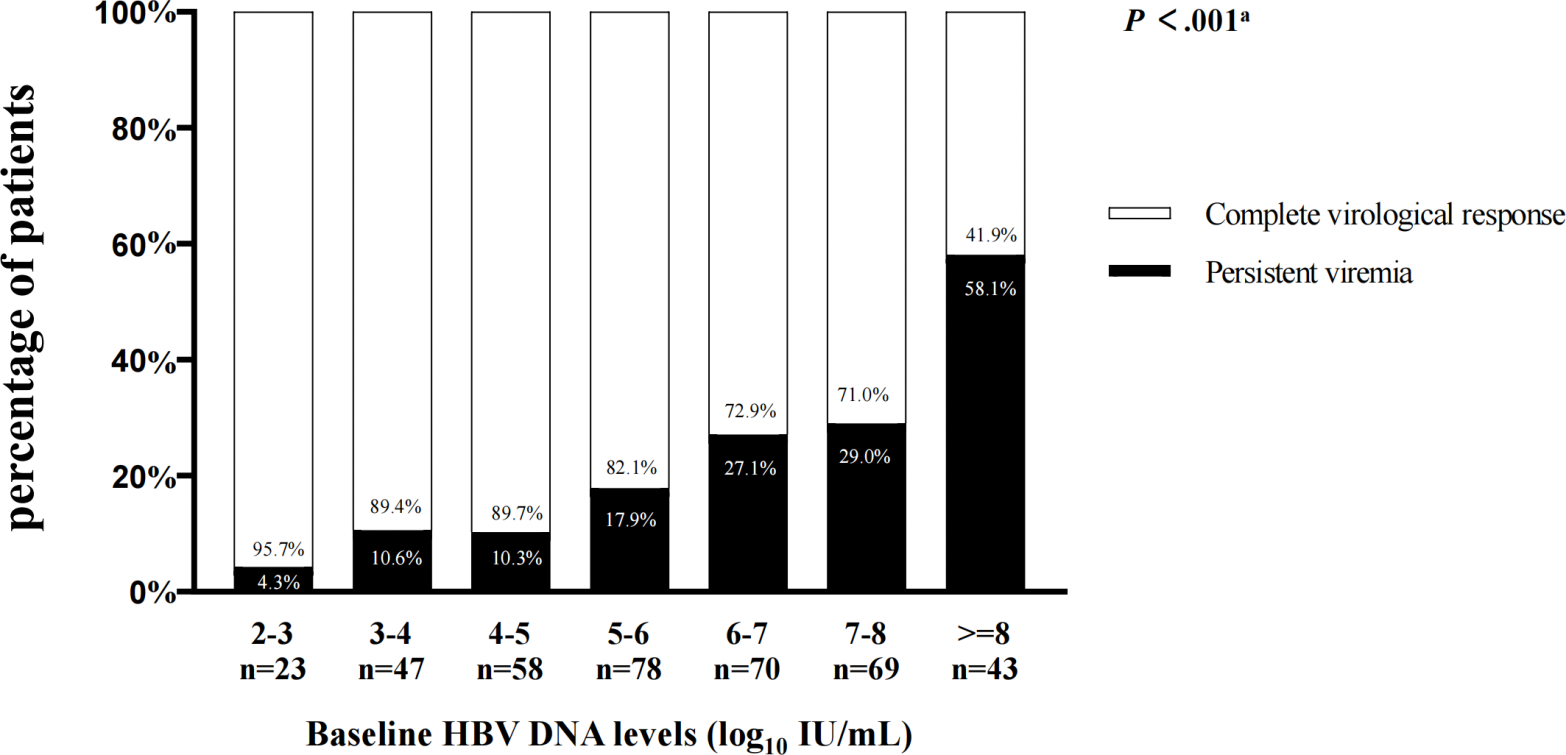
The virological responses in patients after 78-week antiviral therapy according to different HBV DNA levels at baseline. ^a^Comparisons by the Chi-squared test (between 2-3, 3-4, 4-5, 5-6, 6-7 ,7-8, and ≥8 groups).

**Figure 2:**
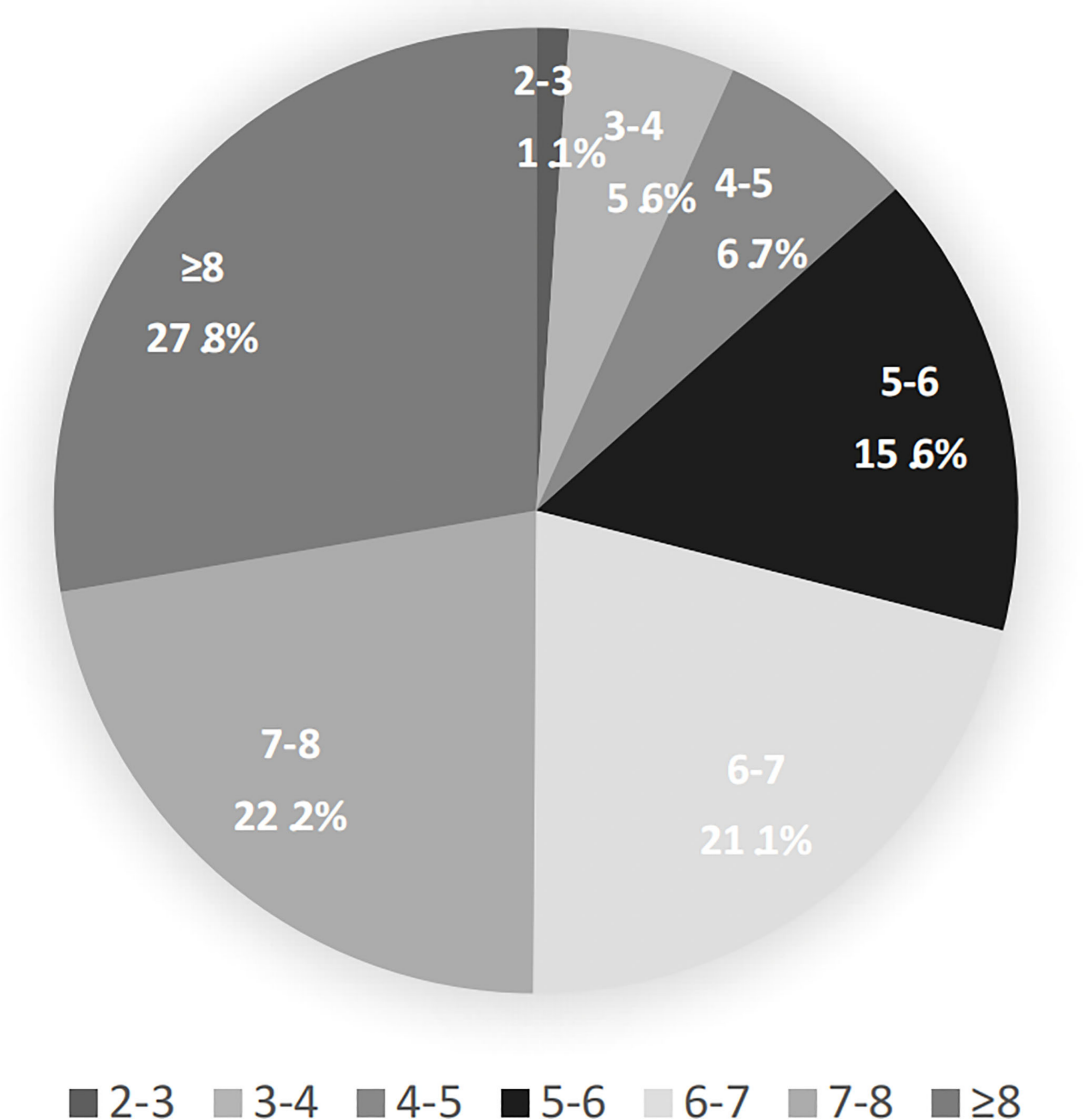
The proportion of patients in persistent viremia group according to different HBV DNA levels at baseline.

Furthermore, according to different baseline HBV DNA levels, we analyzed the virological responses in all patients after 78-week antiviral therapy (**Figure 1**). Among patients with baseline HBV DNA level <2 log_10_ IU/mL, none has showed PV. As baseline HBV DNA levels increased, the proportion of patients with PV also increased after 78 weeks of treatment (when the HBV DNA levels were between 2-3, 3-4, 4-5, 5-6, 6-7,7-8, and ≥8, there were 4.3%, 10.6%, 10.3%, 17.9%, 27.1%, 29.0% and 58.1% patients occurred PV, respectively). Similarly, in patients with PV, the higher the baseline HBV DNA level was, the greater the proportion of patients has developed PV (when the HBV DNA levels were between 2-3, 3-4, 4-5, 5-6, 6-7, 7-8, and ≥8, the proportions were 1.1%, 5.6%, 6.7%, 15.6%, 21.1%, 22.2% and 27.8%, respectively.) (**Figure 2**).

**Figure 3:**
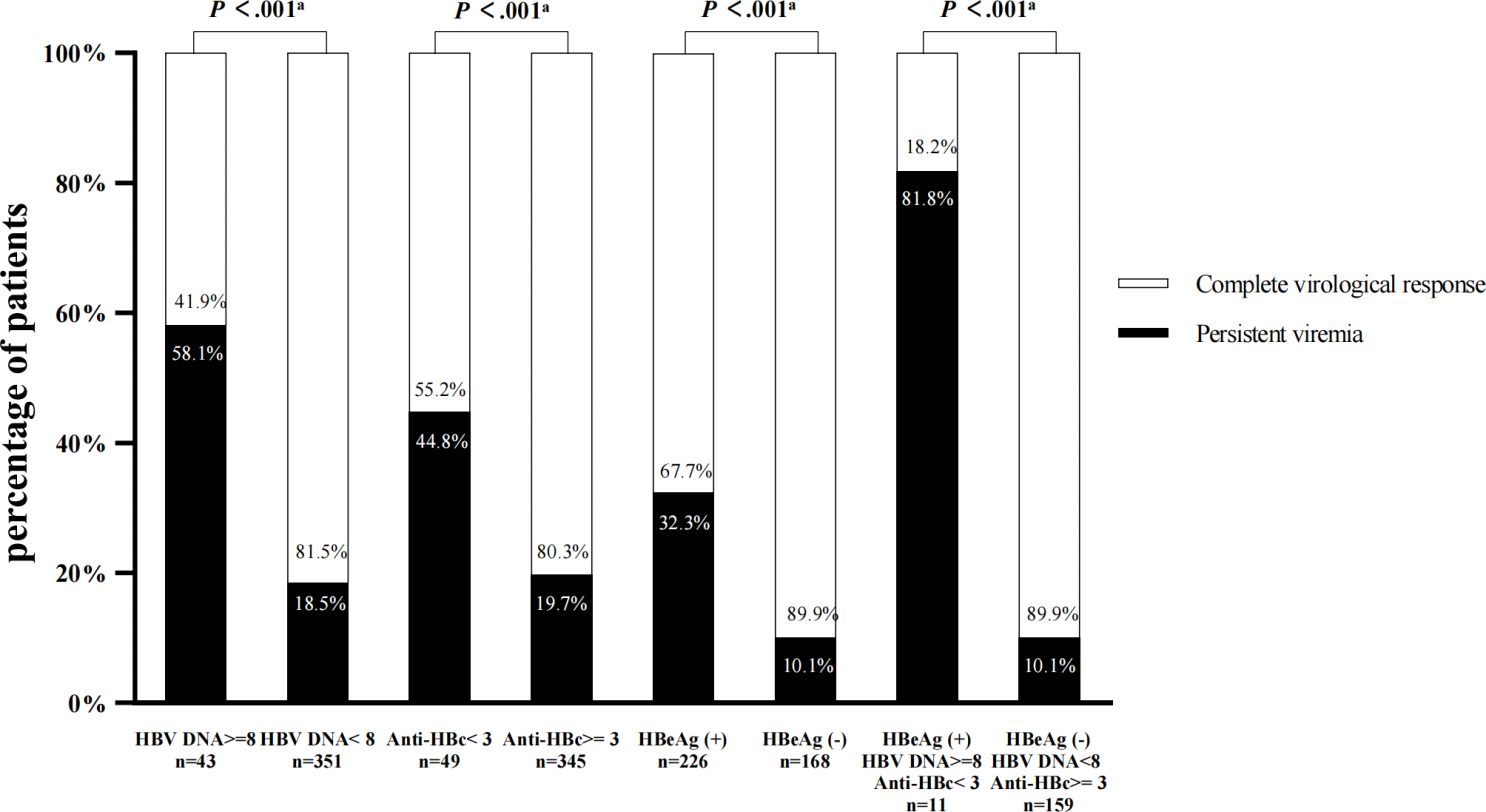
Effects of baseline HBV DNA level ≥8 log10 IU/mL, Anti-HBc level<3 log10 IU/mL and HBeAg status on persistent viremia at week 78. ^a^Comparisons between the related two groups (HBV DNA level ≥8 log10 IU/mL vs <8 log10 IU/mL; Anti-HBc level<3 log10 IU/mL vs ≥3log10 IU/mL; HBeAg+, HBV DNA level ≥8 log10 IU/mL and Anti-HBc level<3 log10 IU/mL vs HBeAg- , HBV DNA level<8 log10 IU/mL and Anti-HBc level ≥3 log10 IU/mL ).

In a multivariate analysis (**Table 2**), 3 baseline variables were identified to be associated independently with PV at week 78: baseline HBV DNA level ≥8 log_10_ IU/mL (odds ratio [OR], 3.727; 95% confidence interval [CI], 1.851-7.505; *P*<0.001), baseline Anti-HBc level<3 log_10_ IU/mL (OR, 2.384; 95% CI, 1.223-4.645; *P*=0.011), and baseline HBeAg seropositivity (OR, 2.871; 95% CI, 1.563-5.272; *P*<0.001). As shown in **Table 2**, among patients with baseline HBV DNA level ≥8 log_10_ IU/mL, 58.1% of patients had PV at week 78 compared with 18.5% of those with CVR. Similarly, 44.8% of patients with Anti-HBc level<3 log_10_ IU/mL had PV at week 78 compared with 19.7% of those with CVR. In HBeAg+ patient, 32.3% had persistence viremia and 10.1% achieved CVR at week 78 (both *P*<0.001). Among HBeAg+ patients with HBV DNA level ≥8 log_10_ IU/mL and Anti-HBc level<3 log_10_ IU/mL at baseline, 81.8% had PV at week 78 compared with 10.1% of HBeAg- patients with HBV DNA level<8 log_10_ IU/mL and Anti-HBc level ≥3 log_10_ IU/mL (*P*<0.001) (**Figure 3**). Of the 11 HBeAg-positive patients with baseline HBV DNA level ≥8 log_10_ IU/mL and Anti-HBc level<3 log_10_ IU/mL, none had shown fibrosis progression at week 78 of treatment (*P*<0.001).

**Table 2:** Baseline characteristics associated with persistent viremia (multivariate analysis).

Parameters	OR	95% CI	P value
HBV DNA level ≥8 log10 IU/mL	3.727	1.851-7.505	**<0.0001**
Anti-HBc level<3 log10 IU/mL	2.384	1.223-4.645	0.011
HBeAg positive vs negative	2.871	1.563-5.272	0.001
Age (years) ≤30y			0.241
HBsAg ≥4 log10 IU/mL			0.402
Fibrosis stages >3			0.106

OR, odds ratio; CI, confidence interval; HBV, hepatitis B virus; Anti-HBc, hepatitis B core antibody; HBeAg, hepatitis B e antigen. The meaning of the bold values is to highlight statistically significant indicators.

### On-treatment characteristics associated with persistent positive in HBV DNA level

On-treatment serum immunological, biochemical, and histologic responses associated with PV patients are summarized in **Table 3**. Compared with patients with CVR, patients with PV were less likely to achieve HBeAg loss (21.9% vs 35.9%; *P*=0.034); however, no differences in the reduction of HBsAg level were shown. Significant differences in histologic responses were observed, where patients with PV were less likely to have a worse LSM value (0% vs 5.9%, *P*=0.038) and Ishak fibrosis score (11.1% vs 21.4%, *P*=0.029) than patients with CVR at week 78. In addition, there was no significant difference between the two groups in obtaining normal ALT levels (92.2% vs 91.4%, *P*=0.816) and histological improvement (58.9% vs 50.3%, *P*=0.153) after 78-week antiviral therapy.

**Table 3:** Different treatment responses between persistent viremia group and complete virological response group (univariate analysis).

	Total n=394	Serum HBV DNA level at week 78		P-value*
		Persistent viremia (n=90)	Complete virological response (n=304)	
Serum immunological responses				
HBeAg loss at week 78, n/N (%)	71/226 (31.4)	16/73 (21.9)	55/153 (35.9)	**0.034**
HBsAg level decrease at week 78, n/N (%)	155/394 (68.6)	28/90 (31.1)	107/304 (35.2)	0.473
>1 log decline in qHBsAg, n/N (%)	38/394 (9.6)	13/90 (14.4)	25/304 (8.2)	0.079
Biochemical response				
ALT normal at week 78, n/N (%)	361/394 (91.6)	83/90 (92.2)	278/304(91.4)	0.816
>1 log decline in qAnti-HBc, n/N (%)	194/394 (49.2)	52/90 (57.8)	142/304 (46.7)	*0.065*
Histologic responses				
APRI score progression at weei 78, n/N (%)	4/394 (1.0)	0/90 (0)	4/304 (1.3)	0.620
FIB-4 score progression at weei 78, n/N (%)	34/394 (8.6)	9/90 (10)	25/304 (8.2)	0.598
LSM value progression at week 78, n/N (%)	18/394 (4.6)	0/90 (0)	18/304 (5.9)	**0.038**
Fibrosis progression at week 78, n/N (%)	75/394 (19.0)	10/90 (11.1)	65/304 (21.4)	**0.029**
Inflammation improvement at week 78, n/N (%)	247/394 (62.7)	58/90 (64.4)	189/304 (62.2)	0.695
Histological improvement at week 78, n/N (%)	206/394 (52.3)	53/90 (58.9)	153/304 (50.3)	0.153

ALT, Alanine transaminase; HBV, hepatitis B virus; Anti-HBc, hepatitis B core antibody; HBeAg, hepatitis B e antigen; HBsAg, hepatitis B surface antigen; LSM, liver stiffness measurement. The meaning of the bold values is to highlight statistically significant indicators. *P value provided in Tables 3, 4: comparison between patients with and without complete virological response at week 78.

Furthermore, we divided the patients with fibrosis progression into 3 groups based on baseline Ishak fibrosis score: group 1 with Ishak fibrosis score between 0-2 (33 patients); group 2 with Ishak fibrosis score between 3-4 (39 patients); group 3 with Ishak fibrosis score between 5-6 (3 patients). There were 5 (15.2%), 5 (12.8%) and 0 (0%) patients in groups 1, 2 and 3, respectively, with PV (**Supplementary Figure 2**).

### Estimated risk of HCC occurrence using different HCC risk scores

Considering that some models evaluating the risk of HCC development which suitable for the untreated patients with chronic HBV infection include the serum HBV DNA level as a constituent variable, we selected the models which suitable for the treated patients to avoid overestimating the incidence of HCC. According to the CAMD score (Hsu et al., 2018), more patients with PV were observed in the low-risk group (CAMD score, <8; 73.3% vs 56.9%), whilst less patients with PV were in the high-risk group (CAMD score, >13; 4.4% vs 8.9%), compared to the patients with CVR (*P* =0.018). Although there was no significant difference according to the AMAP score (Fan et al., 2020), AASL-HCC score (Yu et al., 2019), HCC-ESCAVT score (Lim et al., 2020), CAMPA score (Lee et al., 2020), HCC-RESCUE score (Sohn et al., 2017), and mPAGE-B HCC score (Kim et al., 2018), more patients with PV were in the low-risk group than patients with CVR (**Table 4**).

**Table 4:** Risk of HCC development in 394 patients with and without complete virological response at different HCC risk prediction models.

Variables	Total n=394	Persistent viremia n=90	Complete virological response n=304	P-value*
CAMD score				**0.018**
low-risk group, n(%)	239 (60.7)	66 (73.3)	173 (56.9)	
intermediate-risk group, n(%)	124 (31.5)	20 (22.2)	104 (34.2)	
high-risk group, n(%)	31 (7.9)	**4 (4.4)**	**27 (8.9)**	
AMAP score				0.204
low-risk group, n(%)	162 (41.1)	43 (47.8)	119 (39.1)	
intermediate-risk group, n(%)	184 (46.7)	40 (44.4)	144 (47.4)	
high-risk group, n(%)	48 (12.2)	**7 (7.8)**	**41 (13.5)**	
AASL-HCC score				0.091
low-risk group, n(%)	177 (44.9)	48 (53.3)	129 (42.4)	
intermediate-risk group, n(%)	195 (49.5)	40 (44.4)	155 (51.0)	
high-risk group, n(%)	22 (5.6)	**2 (2.2)**	**20 (6.6)**	
HCC-ESC_AVT_ score				0.424
low-risk group, n(%)	281 (71.3)	**69 (76.7)**	**212 (69.7)**	
intermediate-risk group, n(%)	100 (25.4)	19 (21.1)	81 (26.6)	
high-risk group, n(%)	13 (3.3)	2 (2.2)	11 (3.6)	
CAMPAS score				0.538
low-risk group, n(%)	110 (27.9)	**29 (32.2)**	**81 (26.6)**	
intermediate-risk group, n(%)	151 (38.3)	31 (34.4)	120 (39.5)	
high-risk group, n(%)	133 (33.8)	30 (33.3)	103 (33.9)	
HCC-RESCUE score				0.111
low-risk group, n(%)	264 (67.0)	68 (75.6)	196 (64.5)	
intermediate-risk group, n(%)	104 (26.4)	19 (21.1)	85 (28.0)	
high-risk group, n(%)	26 (6.6)	**3 (3.3)**	**23 (7.6)**	
mPAGE-B HCC score				0.679
low-risk group, n(%)	254 (64.5)	**60 (66.7)**	**194 (63.8)**	
intermediate-risk group, n(%)	138 (35.0)	30 (33.3)	108 (35.5)	
high-risk group, n(%)	2 (0.5)	0 (0)	2 (0.7)	

HCC, hepatocellular carcinoma; CAMD, cirrhosis, patient age, male sex, and diabetes; AMAP, age, male, albumin-bilirubin, platelets; AASL, age, albumin, sex, liver cirrhosis; ESC, e antigen seroclearance; AVT, antiviral therapy; HCC-RESCUE, HCC-Risk Estimating Score in CHB patients Under Entecavir; mPAGE-B, modified PAGE-B; CAMPAS, cirrhosis on ultrasonography, age, male gender, platelet count, albumin and liver stiffness. The meaning of the bold values is to highlight statistically significant indicators. *P value provided in Tables 3 and 4: comparison between patients with and without complete virological response at week 78.

## Discussion

In the era of NAs therapy, most patients with CHB achieve CVR and the majority experience fibrosis regression including the reversal of cirrhosis (Chang et al., 2006; Lai et al., 2006; Chang et al., 2010; Xu et al., 2015). However, despite these highly beneficial outcomes, a small number of patients still fail to achieve CVR, and the clinical significance of a PV with respect to the outcome of CHB is not clear. In this analysis of predictive factors for PV after 78 weeks of ETV treatment in a well-characterized cohort of CHB patients, we found that HBV DNA level ≥8 log_10_ IU/mL, Anti-HBc level<3 log_10_ IU/mL, and HBeAg seropositivity are independently predictive of failure to achieve CVR. Furthermore, of the 11 HBeAg-positive patients with HBV DNA level ≥8 log_10_ IU/mL and Anti-HBc level<3 log_10_ IU/mL at baseline, 9 (81.8%) had persistent positive in HBV DNA level and 0 had fibrosis progression at week 78 of treatment. Of the 90 patients with PV, only 10 (11.1%) had fibrosis progression and 0-7 (0-7.8%) were with high risk of HCC occurrence according to different HCC risk scores.

Higher levels of HBV DNA are associated with an increased risk for HCC and cirrhosis (Chen et al., 2011), but its impact on virological response is debatable. Yuen et al. reported that 100% of treatment-naïve HBV patients who received entecavir for 3 years with baseline HBV DNA <8 log_10_ copies/mL had undetectable HBV DNA (<12 IU/mL), whereas only 75% of patients with baseline HBV DNA ≥8 log_10_ copies/mL did (Yuen et al., 2011). Gordon et al. reported that equal virologic responsiveness between CHB patients with high viral load (HVL) (HBV DNA ≥9 log_10_ copies/mL) and with non-HVL, 98.3% of HVL and 99.2% of non-HVL patients achieving HBV DNA <400 copies/mL by week 240 (Gordon et al., 2013). Chan et al. reported that 55% of HBeAg-positive patients with high levels of HBV DNA (mean baseline level of HBV DNA of 8.41 log_10_ IU/mL) and normal levels of ALT treated with TDF had levels of HBV DNA <69 IU/mL at week 192 (Chan et al., 2014). In our analysis, only 77.2% of treatment-naïve CHB patients who received entecavir for 78 weeks achieve CVR, 81.5% of patients with baseline HBV DNA <8 log_10_ IU/mL achieved CVR (HBV DNA <20 IU/mL), whilst the CVR rates were only 41.9% in patients with baseline HBV DNA ≥8 log_10_ IU/mL. The poor efficacy of ETV may be related to previous using of lamivudine (LAM) and telbivudine (LdT), but the proportion of such patients was similar between the two groups. This finding may be attributed to the short time courses of antiviral therapy or some patients may be in immune-tolerant phase. Larger longitudinal studies are warranted to explore this factor further and its potential effect on the virological response to antiviral treatment in CHB patients.

Previous studies have shown that rates of CVR in clinical trials of CHB patients were different between HBeAg+ and HBeAg- patients, and the former tended to have lower rates of CVR compared to the latter (Chang et al., 2006; Lai et al., 2006; Gish et al., 2007; Pan et al., 2012; Marcellin et al., 2013). Consequently, the observed associations between HBeAg-positive and a PV state is not entirely surprising. Among 875 treatment-naive chronic hepatitis B virus (HBV) mono-infected patients, 377 patients with low-level viremia (LLV; <2,000 IU/mL), Kim et al. reported that HBeAg status was the only significant factor associated with LLV (Kim et al., 2017), which coincided with our results.

Our finding that Anti-HBc level at baseline is associated independently with failure to achieve CVR on NAs therapy is novel and may relate to the intensity of liver inflammation. It remains controversial of the association between ALT levels and liver inflammation. Some studies have suggested that substantial CHB patients with normal ALT levels exhibit severe liver damage. Chao et al. reported that approximately one fifth (20.7%) of CHB patients with ALT ≤ 40 IU/L may have significant hepatic fibrosis. The corresponding proportion was 27.8% even when the newer ULN of 30 IU/L (males) and 19 IU/L (females) was applied (Chao et al., 2014).

Recent studies proposed that serum anti-HBc levels could serve as a promising marker for predicting the severity of liver inflammation and exhibited a high diagnostic accuracy in CHB patients with normal ALT (Song et al., 2015; Zhou et al., 2017). In that studies, Anti-HBc can accurately reflect the inflammation of the liver. In our analysis, the levels of ALT at baseline were comparable between the two groups, but the level of Anti-HBc in patients with PV was lower than patients in CVR (3.6 vs 3.9 log_10_ IU/mL, *P*<0.001), which indicated that Anti-HBc is a more responsive indicator of liver inflammation than ALT. Of 49 patients with Anti-HBc level<3 log_10_ IU/mL, 22 (44.9%) failed to achieve CVR, which suggested that some of patients with low levels of Anti-HBc may be in the immune tolerance phase.

Evidence has emerged that incomplete virologic suppression, particularly intrahepatic viral transcriptional activity, was associated with abnormal liver histopathology in a cross-sectional study (Wang et al., 2017). Sun et al. suggested that detectable low-level HBV DNA was associated with fibrosis progression in patients with chronic HBV infection during 78 weeks of entecavir therapy (Sun et al., 2020). But in our study, patients with PV were less likely to have a worse LSM value (0% vs 5.9%, *P*=0.038) and fibrosis progression (11.1% vs 21.4%, *P*=0.029) than patients with CVR during 78 weeks of entecavir therapy. Furthermore, Patients with PV were more likely to have a low-risk of HCC development than those with CVR. High viral load (HVL) (HBV DNA≥8 log_10_ copies/mL), positive HBeAg, slightly liver inflammation (Anti-HBc <3 log_10_ IU/mL), low-risk of fibrosis progression and HCC development, all these suggested that these particular PV patients may be in the immune tolerance phase (Sarin et al., 2016; European Association for the Study of the Liver, 2017; Terrault et al., 2018). Even with potent drugs like entecavir, their virological response was unsatisfactory. Hence, the initiation of antiviral therapy could be delayed or waiting for stronger and more effective drugs may be alternative choice. Further prospective studies with larger sample size and longer follow-up are needed to confirm this finding and may provide some hint to optimization of histological and longterm clinical outcomes of antiviral therapy.

Our study has several limitations. First, the definition of PV was based on a single serum HBV DNA measurement at week 78. Although HBV DNA was measured at week 0, 26, 52 and 78 in local hospitals, respectively, we selected week 0 and 78 data detected at central laboratory to ensure the consistency of the results. Second, the follow-up time was relatively short. There was a possibility that PV observed at week 78 may turn to CVR after longer follow-up. Third, the development of drug-associated mutations was not investigated. However, considering the good compliance of patients and the extremely low resistance rate of entecavir, HBV mutations are unlikely to be the main cause of PV during antiviral therapy. Finally, our study used only ETV as a first choice for treatment of naïve CHB patients. Other NAs may have differential results. Therefore, the result of present study applies only to the patients using ETV.

In conclusion, our study suggested that HBV DNA level ≥8 log_10_ IU/mL, Anti-HBc level<3 log_10_ IU/mL and HBeAg seropositivity at baseline contributed to persistent positivity in HBV DNA level in patients with CHB receiving 78-week antiviral treatment. The risk of HCC development and the rate of fibrosis progression in these patients were low. Maybe these particular PV patients were in the immune tolerance phase, and the initiation of antiviral therapy could be delayed or waiting for stronger and more effective drugs may be another choice. Further well-designed prospective studies with large-scale and longer follow-up are needed to confirm this finding and may provide some hint to judge true immune tolerance phase.

## Data availability statement

The original contributions presented in the study are included in the article/**Supplementary Material**. Further inquiries can be directed to the corresponding authors.

## Ethics statement

The study was approved by the Ethics Committee of Peking University First Hospital and other 23 teaching hospitals. The patients/participants provided their written informed consent to participate in this study.

## Author contributions

JL, X-QD, HZ and G-QW designed the experiments; HZ and G-QW provided the overall principle and direction of the study; X-QD and JL gathered and analyzed data, and drafted the manuscript; X-QD, Y-QL, and CZ done the laboratory examination. L-HC, Z-QZ, W-FZ, Q-HS, D-ZZ, A-LM, QX, H-LG, GZ, Y-XL, JS, S-BX and China HepB Related Fibrosis Assessment Research Group had participated in acquisition of data, revision of the manuscript for important intellectual content. All authors contributed to the article and approved the submitted version.

## Glossary

**Table:**

HBV	Hepatitis B Virus
CHB	chronic hepatitis B
HCC	hepatocellular carcinoma
CVR	complete virological response
PV	persistent viremia
NAs	Nucleos(t)ide analogues
TDF	Tenofovir disoproxil fumarate
ETV	entecavir
HBeAg	hepatitis B e antigen
CLD	chronic liver diseases
ALT	alanine aminotransferase
cccDNA	covalently closed circular DNA
INR	international normalized ratio
PLT	platelet counts
TBil	total bilirubin
AFP	alpha fetoprotein
Anti-HBc	anti-hepatitis B virus core antibody
KPa	kilopascals
IQR	interquartile range
FIB-4	Fibrosis-4
APRI	AST to platelet ratio index
HAI	histology activity index
OR	odds ratio
CI	confidence interval
LLV	low-level viremia
HVL	High viral load
BMI	body mass index
CR	creatinine
FPG	fasting plasma glucose
LSM	liver stiffness measurement
HBsAg	hepatitis B surface antigen
HBeAg	hepatitis B e antigen
HBV	hepatitis B virus
ULN	upper limit of normal
CAMD	cirrhosis, patient age, male sex, and diabetes
AMAP	age, male, albumin-bilirubin, platelets
AASL	age, albumin, sex, liver cirrhosis
ESC	e antigen seroclearance
AVT	antiviral therapy
HCC-RESCUE	HCC-Risk Estimating Score in CHB patients Under Entecavir
mPAGE-B	modified PAGE-B
CAMPAS	cirrhosis on ultrasonography, age, male gender, platelet count, albumin and liver stiffness
