# Supplementary figures and images for: Factors associated with persistent positive in HBV DNA level in patients with chronic Hepatitis B receiving entecavir treatment
Source: Front Cell Infect Microbiol. 2023 Jun 16;13:1151899. doi: 10.3389/fcimb.2023.1151899 (PMC10311917; doi:10.3389/fcimb.2023.1151899)

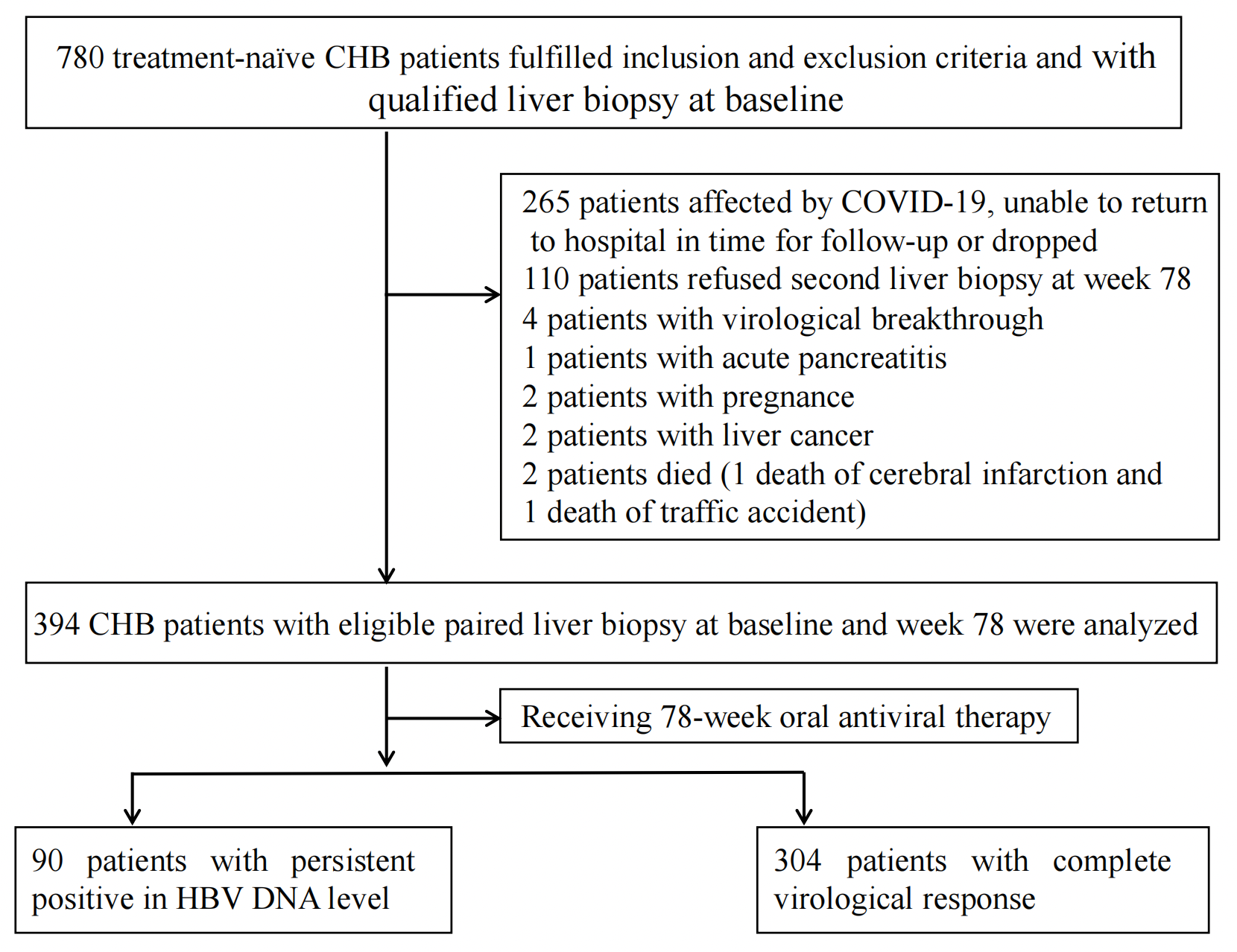

Supplement: Supplementary file 1 [file Image_1.jpeg]

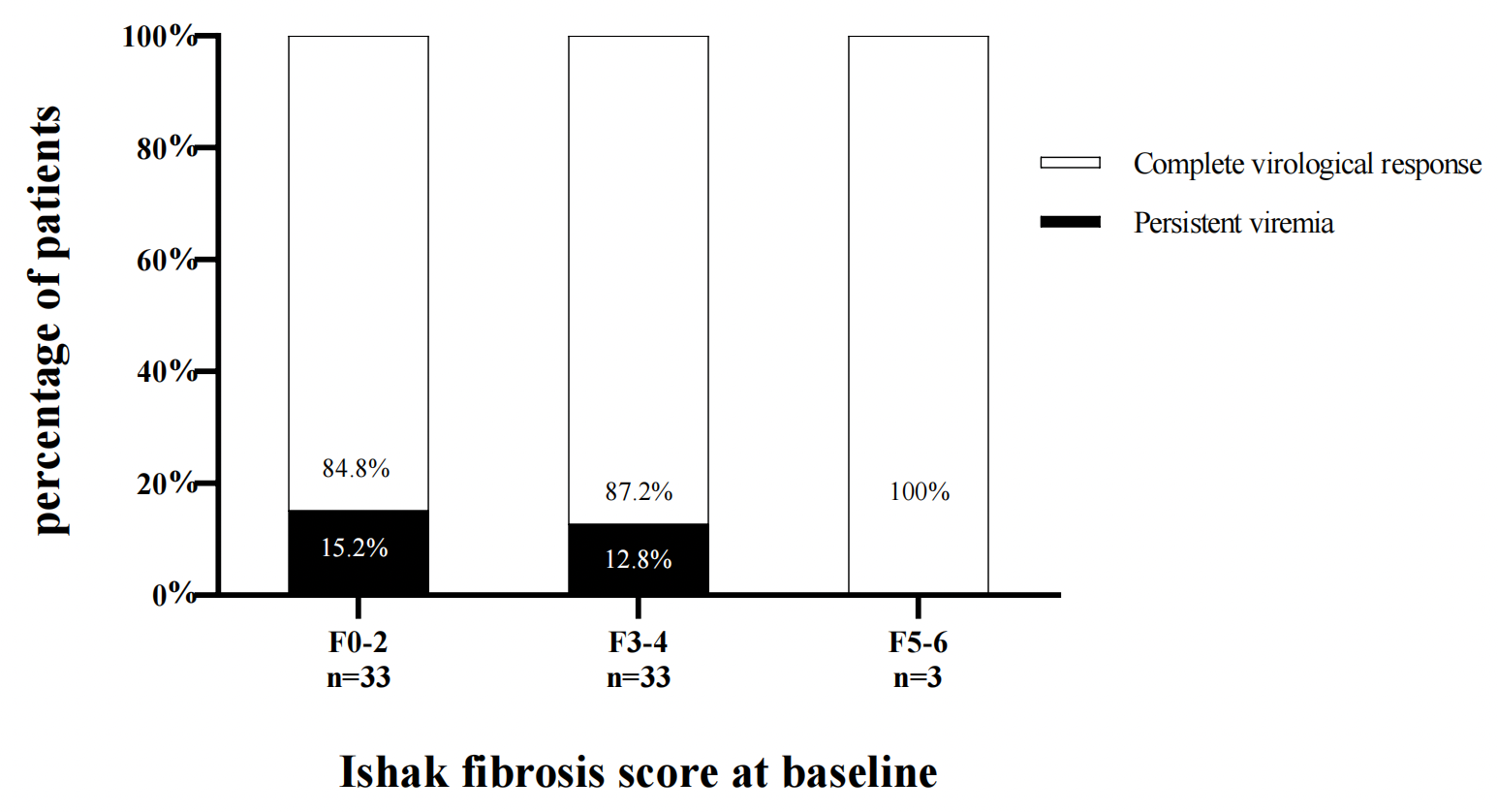

Supplement: Supplementary file 2 [file Image_2.jpeg]
